# Cognitive flexibility training for chronic pain: a randomized clinical study

**DOI:** 10.1097/PR9.0000000000001120

**Published:** 2024-02-12

**Authors:** Katherine J. Holzer, Marko S. Todorovic, Elizabeth A. Wilson, Aaron Steinberg, Michael S. Avidan, Simon Haroutounian

**Affiliations:** a Department of Anesthesiology, Washington University School of Medicine, St. Louis, MO, USA; b Department of Anesthesiology, Virginia Mason Medical Center, Seattle, WA, USA; c Emergency Department, SSM Health St. Mary's Hospital, St. Louis, MO, USA

**Keywords:** Cognitive flexibility, Neurocognitive training, Pain severity, Pain interference

## Abstract

**Introduction:**

Previous studies suggest an association between cognitive flexibility and development of chronic pain after surgery. It is not known whether cognitive flexibility can be improved in patients with chronic pain.

**Objectives:**

This study tested whether a neurocognitive training program results in improved cognitive flexibility and pain in patients with chronic pain.

**Methods:**

We conducted a single-center, prospective, randomized study investigating 5-week daily neurocognitive training in patients with chronic pain. Participants (n = 145) were randomized into neurocognitive training or care as usual, and they completed assessments at baseline, posttreatment, and 3 months. The treatment group was asked to spend 35 minutes daily completing a program with tasks on cognitive flexibility, memory, attention, and speed. The primary outcome was performance on the neurocognitive performance test (NCPT). Secondary outcomes included levels of pain interference and severity.

**Results:**

At 5 weeks, the treatment group showed greater improvements on NCPT compared with the control group (*d* = 0.37); effect size was smaller at 3 months (*d* = 0.18). The treatment group reported lower pain severity at 5 weeks (*d* = 0.16) and 3 months (*d* = 0.39) than the control group, but pain interference was only lower at 3 months (*d* = 0.20).

**Conclusions:**

Outcomes suggest that using neurocognitive training to modify cognitive flexibility in patients with chronic pain may improve pain severity. This study provided effect size estimates to inform sample size calculations for randomized controlled trials to test the effectiveness of neurocognitive interventions for the prevention and treatment of chronic pain.

## Abstract 2

Supplemental Digital Content is Available in the Text.

Participating in a neurocognitive training program may improve overall cognitive performance and reduce pain severity over 3 months in individuals with chronic pain.

## 1. Introduction

Cognitive flexibility is defined as the ability to make behavioral adjustments in the face of changing external stimuli,^26^ such that one is able to shift their attention from their current task in response to a new stimulus or focus on a new cognitive task while experiencing ongoing pain. Attal et al.^5^ found that patients who had poorer preoperative performance on neurocognitive measures of cognitive flexibility had a greater prevalence or severity of clinically meaningful persistent pain after surgery, suggesting that impaired cognitive flexibility may be a risk factor for chronic pain. To further quantify the role of cognitive flexibility in the development of chronic pain after surgery, Vila et al.^76^ enrolled a cohort of surgical patients to determine the association between preoperative cognitive flexibility (assessed using the Color-Word Matching Stroop Test [CWMST]) and the incidence of clinically meaningful surgical site pain 6 months postoperatively. Results revealed that poor preoperative performance on the CWMST was associated with higher risk of persistent postsurgical pain.

Cognitive and emotional processes governing adaptations to pain play an important role in the transition from acute to chronic pain.^65,71,72,73,74^ According to the well-established cognitive appraisal of resilience model,^54^ a cognitive appraisal occurs after an adverse/noxious event, and cognitive flexibility is a key contributor to a positive appraisal and subsequently positive adaptation and favorable outcome (vs a maladaptive response and pain persistence). Cognitive flexibility is also associated with greater tolerance of uncertainty when unexpected or adverse events occur.^76^ These data suggest an association between impaired cognitive flexibility and increased risk, or vulnerability, to persistent pain. This premise is also supported by neuroimaging data demonstrating that cortical areas with key roles in pain processing and control^4,7,8,17,19,39,63,75,77–80^ are actively involved in cognitive flexibility tasks.^27,36,44^ Individuals with higher levels of pain may perform worse on tests of cognition^3,32,55,70^; conversely, impaired cognitive flexibility may precede chronic pain development.^5,76^

Despite evidence suggesting a link between cognitive flexibility and the development of chronic surgical-site pain, it is not known whether cognitive flexibility can be improved in patients with chronic pain, and whether this improvement would translate to reduction in pain. For example, interventions focusing on the cognitive processing of pain, including cognitive behavioral therapy and mindfulness-based approaches, can provide long-lasting pain relief.^25,28,29,34,35,73,74^ However, it is unclear whether these interventions alleviate pain through modifying cognitive flexibility or other components of executive function (EF),^20,48^ such as self-inhibition, emotional control, and working memory.

A computer-based approach for neurocognitive training has the potential to provide a scalable standardized method to modify cognitive flexibility with objective, measurable endpoints for subject performance and training adherence. Computerized cognitive flexibility training has led to improved performance on measures of cognitive flexibility and other EF tests in patients with anorexia nervosa and posttraumatic stress disorder.^10,16,21,37^ Furthermore, chronic pain patients appear to be amenable to improvements in EF performance and self-reported cognition through a computer-based intervention.^6^

In this prospective study, we assessed a neurocognitive intervention for patients with chronic hip, back, and knee pain. The primary goal was to understand whether a 5-week training program could boost cognitive function in these individuals. Simultaneously, the secondary objective was to assess the program's effect on pain severity and interference, leading to our dual hypotheses that computer-based cognitive training over 5 weeks (1) can improve cognitive flexibility scores in the context of chronic pain (primary outcome) and (2) can improve pain severity and pain interference scores in individuals with chronic pain (secondary outcome).

## 2. Methods

### 2.1. Study overview

This was a single-center, prospective, randomized, parallel-group clinical study. This study was approved by the Institutional Review Board of Washington University School of Medicine in St. Louis and registered on clinicaltrials.gov (https://clinicaltrials.gov/ct2/show/NCT03398408).

### 2.2. Participants

Study coordinators recruited participants from the Washington University Pain Center and from the community. Inclusion criteria were as follows: (1) adults between 18 and 70 years with chronic (>3-month duration) hip, knee, or back pain; (2) documented moderate-to-severe chronic pain (eg, a physician's note or visit summary), defined as pain greater than or equal to 4 on a 0–10 numerical rating scale; (3) English fluency; and (4) access to an email account. Exclusion criteria were as follows: (1) lack of basic computer skills/no access to a computer with internet; (2) diagnosed Alzheimer disease or documented severe cognitive impairment; (3) severely impaired vision or color blindness; (4) inability to complete cognitive testing; (5) an interventional pain procedure within one week before enrollment; or (6) scheduled to undergo an interventional or surgical procedure during the study period. Eligibility was not affected by the participants' current pain treatment, whether pharmacological or nonpharmacological. Participants provided their written informed consent before completing the baseline questionnaires, which were conducted during the in-person enrollment visit. Participants were paid up to $45 for their participation ($15 for baseline questionnaire completion and $15 each for completion of 5-week and 3-month assessments).

### 2.3. Randomization

Participants were randomized by the study team. Randomization was done in a 2:1 ratio, in blocks of 6, using an online research randomizer (https://www.randomizer.org/). Twenty-five sets of 6 unique numbers were randomly generated from the sets 1 to 6. Two numbers were randomly selected to represent the control condition, and 4 numbers were randomly selected to represent intervention. Study coordinators then assigned participants to the intervention or control condition depending on their assigned number. Research coordinators were required to provide logistical guidance for patients engaging in the training program; therefore, group assignment was not concealed from the study team. Each participant was provided with a unique link to their computerized tests. Only participants in the intervention group had access to the neurocognitive training module.

### 2.4. Description of intervention

Patients in the intervention group continued their care as usual and were assigned to daily neurocognitive training, which they completed at home. The training consisted of completing predetermined modules (games) on the Lumos Labs platform. There were 40 games in total, and participants could play 10 games per day, which appeared in a preset order. Participants were able to skip games and play each game multiple times per day. Overall, 40% of the neurocognitive training session comprised tasks on cognitive flexibility. The cognitive flexibility subset included the following: *Brain Shift 2* (task switching between numbers vs letters), *Disillusion 2* (task switching between matching shape vs color), *Ebb and Flow* (task switching between shape vs direction of movement), *Robot Factory* (response inhibition; ignoring incorrect cues), *Chalkboard Challenge 2* (numerical estimation), and *Organic Order* (logical reasoning). The other 60% of the time included 20% on memory (*Memory Matrix 2, Tidal Treasures, Pinball Recall*), 20% on attention (*Lost in Migration 2, Train of Thought*), and 20% on speed (*River Ranger, Spatial Speed Match 2*). Participants were asked to complete 35 minutes of training daily, for 5 weeks. This training schedule was selected based on results of a previous study demonstrating that these training modules influenced neurocognitive performance with 966 minutes of average engagement.^38^ The authors of the previous study recommended a target of 20 hours (or 1200 minutes) total as necessary to change neurobiological processes. As a result, we aimed for 1225 minutes total (35 minutes per day for 35 days or 5 weeks). Participants were not restricted from continuing to play the cognitive games after study completion.

### 2.5. Description of control

Participants in the control group were not assigned to the neurocognitive intervention of daily training and continued their care as usual.

### 2.6. Measurements

#### 2.6.1. Baseline assessment

The baseline assessment included a detailed medical history, a history of chronic pain and analgesic use, along with the participant's current use of medications which primarily act on the central nervous system (antidepressants, stimulants, etc). Participants completed assessments of pain, catastrophizing, anxiety, and depression. We chose the following measures (Brief Pain Inventory [BPI], Pain Catastrophizing Scale [PCS], Hospital Anxiety and Depression Scale [HADS]) because they are commonly used to describe these characteristics in pain populations.^33^ The BPI^22^ was used to assess pain severity and interference in their daily lives. The pain severity score represents the arithmetic mean of 4 severity items and ranges from 0 to 10. The pain interference score is measured from the arithmetic mean of 7 interference items and ranges from 0 to 10. The BPI has been widely validated in a variety of populations, including individuals with chronic pain.^65,71^ The PCS^68^ has 13 items quantifying 3 components of pain: rumination (scores range 0–16), magnification (0–12), and helplessness (0–24). The total PCS score ranges from 0 to 52, with higher scores indicating higher levels of catastrophizing. The 14-item HADS^79^ was used to assess participants' anxiety and depression over the past week. The anxiety and depression subscores range from 0 to 21. Both the HADS and PCS are recommended for patient characterization in the context of chronic pain.^33^

As in previous studies,^5,76^ changes in cognitive flexibility were measured with the Trail Making Test (TMT) Parts A and B,^14^ and CWMST^80^ with the addition of the Neurocognitive Performance Test (NCPT). Both TMT A and B examine scanning, speed, and motor responses, whereas part B additionally tests the simultaneous maintenance of 2 mental sequences, sustained attention and working memory, and cognitive flexibility.^23–25^ The CWMST has been validated for use in testing EF, particularly the ability to sort out task-relevant information with concurrent distracting information.^26,28^

The TMT and CWMST each took approximately 5 minutes to complete, including instructions. For each test, the participant was given verbal instructions as well as an example for each task. The participant was then asked to complete the task using pencil and paper format. The time required to complete each task was measured by stopwatch. The participant's scores for each measure reflect the number of seconds they took to complete the tasks. The cognitive assessments were administered in the same order for each participant by a trained examiner. Following the enrollment visit, participants were instructed to complete the NCPT within 48 hours of enrollment, before the initiation of the training module. All instructions appeared on the screen as a part of these tests. Neurocognitive performance test is a composite measure of performance on 9 subtests (TMT A, TMT B, digit symbol coding, dual search, go/no-go, grammatical reasoning, progressive matrices, reverse memory span, scale balance) assessing 9 cognitive domains (working memory, visuospatial memory, psychomotor speed, fluid and logical reasoning, response inhibition, numerical calculation, and selective and divided attention). Participants were also asked to complete a computerized CWMST on Lumos Labs platform at the same opportunity. The full description of each subtest of the NCPT batteries are outlined elsewhere.^41^ It has been validated in a sample of >130,000 participants, including test–retest reliability and concurrent validity in >35,000 participants.^49^

The cognitive training intervention took place during the 5 weeks following initial testing.

#### 2.6.2. Follow-up assessment

Participants were reassessed with the computerized CWMST, NCPT (includes the computerized TMT A and TMT B), and BPI within 1 to 3 days after the 5-week training completion and then again 3 months later. Patients received either an email, phone call, or both as a reminder to complete their assessments. Outcome measures were collected remotely in a bias-free manner with no interpretation by the coordinator.

Information regarding medications was extracted from the patient medical record as recorded by nursing staff or physicians, changes to outpatient pain medication regimen, or from the physician report.

### 2.7. Outcomes

The primary outcome of this study was change in computerized NCPT scaled score over 5 weeks of cognitive training between the training group and the control group. Secondary outcomes included the changes from baseline on the BPI pain severity and pain interference subscores. Additional outcomes included changes in NCPT flexibility subscores and computerized CWMST scores from baseline to 5 weeks, as well as all outcomes from baseline to 3 months postintervention follow-up.

The flexibility subscore of NCPT is an arithmetic mean of the flexibility specific NCPT tasks (digit symbol coding, grammatical reasoning, progressive matrices, scale balance, and TMT B). The CWMST interference T-score is calculated by subtracting the predicted color word (PCW) score from the actual CW score.^59^ A T-score of 50 indicates a difference score of 0, and lower scores (<50) suggest deficiencies in performing cognitive flexibility tasks.

To determine the proportion of patients achieving meaningful relief following the intervention, we calculated the proportion of patients achieving 30% or more reduction of pain from baseline, as well as 50% or more reduction in pain from baseline, for both BPI pain severity and pain interference measures. This allowed for the calculation of the number needed to treat (NNT) for the intervention.

### 2.8. Statistical analysis

The arithmetic means, standard deviations, and 95% confidence intervals (CI) for continuous variables, and percentages for categorical variables were calculated for baseline values. Data on BPI and NCPT (flexibility and computerized CWMST scores) at 5 weeks and 3 months were compared between the groups with an unpaired *t* test. Between-group effect sizes were calculated using Cohen *d* ^23^ based on the difference in mean change scores for each cognitive and pain-related outcome between the training and control groups at each time point. Effect sizes of 0.2 to 0.5, 0.5 to 0.8, and 0.8+ were interpreted as small, medium, and large effects, respectively.^23^ To visualize the BPI, NCPT, flexibility NCPT, and CM scores for all subjects over the entire study period, spaghetti plots, each with lines for arithmetic mean score by group, were created.

As a post hoc analysis, we examined differences in scores by adherence in the intervention group only. Changes in outcome measures at 5 weeks and 3 months were compared with paired *t* tests in the intervention group in total and separated by participants with ≥80% adherence (ie, >980 of the total 1225 planned minutes trained) vs <80% adherence.

Because we did not have a priori data on the effect size of the intervention, we decided on a convenience sample of 150 participants in this study (100 in the intervention group and 50 in the control group).

## 3. Results

**Figure 1.**
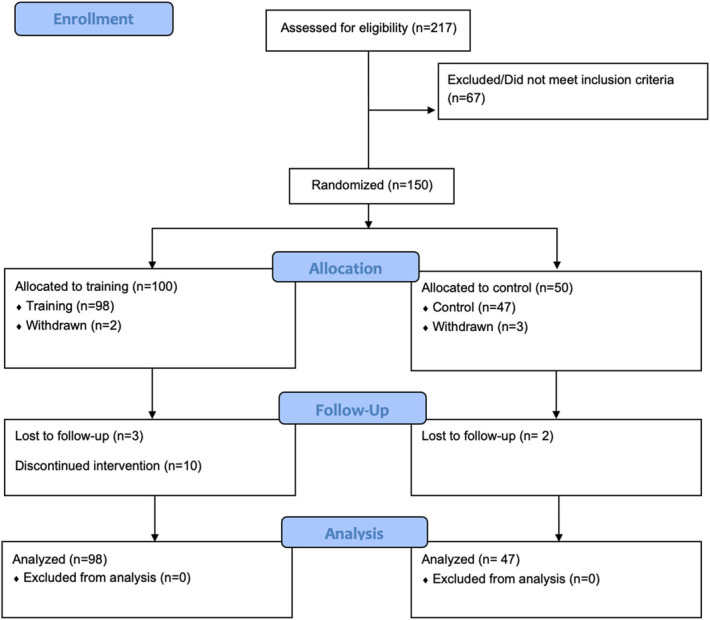
Consolidated Standards of Reporting Trials diagram. Reasons for losses and exclusions after randomization: Participants did not complete any baseline assessment/questionnaires (n = 2 in training group, n = 3 in control group); loss to follow-up (n = 3 in the training group, and n = 2 in the control group); challenges with computer access/skills for training requirements (n = 6, all in training group); and voluntary withdrawal for other reasons (n = 4, all in the training group).

Of the 217 patients screened in the main study cohort, 67 failed eligibility screening, resulting in 150 enrolled and randomized patients (Fig. 1).

### 3.1. Sample characteristics

Table 1 presents demographic information and baseline results for 145 study participants, separated to training vs control. The randomization provided adequate balance across groups with no significant differences in baseline characteristics.

**Table 1:** Demographics and baseline information for study participants.

	All participants (n = 145)	Training (n = 98)	Control (n = 47)	*P*
Age, mean (SD)	54.8 (11.5)	54.5 (11.4)	55.7 (11.9)	0.56
Female sex, %	78.0	75.5	83.0	0.31
Caucasian race, %	71.0	68.4	76.6	0.31
Education, median years (IQR)	16.0 (14.0,18.0)	16.0 (14.0,18.0)	16.0 (14.0,18.0)	0.55
Body mass index, mean (SD)	32.7 (8.6)	33.2 (8.7)	31.6 (8.3)	0.30
Trail making test part A score, median (IQR)	22.7 (18.1–28.4)	21.5 (17.5–28.2)	23.7 (19.2–29.5)	0.72
Trail making test part B score, median (IQR)	47.2 (38.6–63.7)	48.0 (38.5–61.0)	46.5 (39.5–64.9)	0.45
Trail making test part B minus part A, median (IQR)	24.2 (15.4–36.5)	24.5 (15.9–35.2)	22.6 (14.7–38.0)	0.33
CWMST interference T-score, mean (SD)	50.4 (7.1)	50.2 (7.0)	50.6 (7.4)	0.76
Brief pain inventory				
Pain severity score, median (IQR)	4.8 (3.3,6.3)	5.0 (3.3,6.3)	4.5 (3.3,6.0)	0.29
Pain interference score, median (IQR)	4.4 (2.4,6.3)	4.5 (2.4,6.4)	4.4 (2.4,6.1)	0.97
Hospital anxiety and depression scale				
Depression subscore, mean (SD)	4.7 (3.7)	4.6 (3.7)	5.0 (3.8)	0.52
Anxiety subscore, mean (SD)	5.8 (3.8)	5.6 (3.4)	6.3 (4.4)	0.29
Pain catastrophizing scale				
Rumination, mean (SD)	5.9 (4.3)	6.0 (4.3)	5.7 (4.2)	0.71
Magnification, mean (SD)	3.2 (2.7)	3.1 (2.7)	3.3 (2.7)	0.63
Helplessness, mean (SD)	6.6 (4.9)	6.5 (5.0)	6.8 (4.9)	0.80
Total score, mean (SD)	15.7 (10.6)	15.6 (10.7)	15.8 (10.6)	0.93
Baseline comorbidities, %				
Anxiety	30.4	27.6	36.2	0.55
Depression	37.9	35.7	42.6	0.62
Diabetes	13.3	17.5	4.35	0.09
Obstructive sleep apnea	21.0	20.6	21.7	0.76
Baseline medications (yes)				
Anticonvulsants	17.2	18.4	14.9	0.60
Antidepressants	37.9	37.8	38.3	0.95
Anxiolytics and muscle relaxants	24.1	24.5	23.4	0.89
Opioids	22.8	19.4	29.8	0.16
Non-opioid analgesics	60.0	58.2	63.8	0.51
Physical, complementary, and alternative therapies	2.1	2.0	2.1	
Other medications	3.5	3.1	4.3	0.71
Baseline pain location (yes)				
Hip	31.0	30.6	31.9	0.87
Knee	48.3	48.0	48.9	0.91
Low back	65.5	65.3	66.0	0.94

CWMST, Color–Word Matching Stroop test; IQR, interquartile range.

Participants in the intervention group trained an average of 679 ± 297 minutes, which was 55% of the prescribed training amount. The average number of days participants trained during the 5-week period was 33 days.

**Figure 2.**
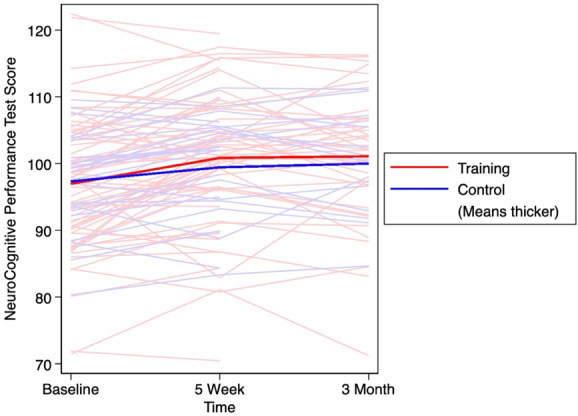
NCPT score over 3 months with thicker lines demonstrating the average score in the training and control groups. NCPT, neurocognitive performance test.

**Figure 3.**
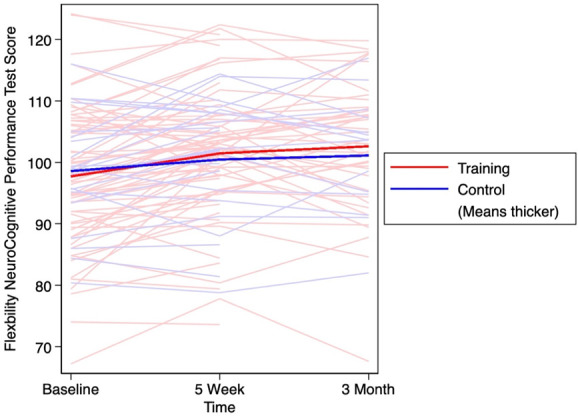
Flexibility NCPT score over 3 months with thicker lines demonstrating the average score in the training and control groups. NCPT, neurocognitive performance test.

**Figure 4.**
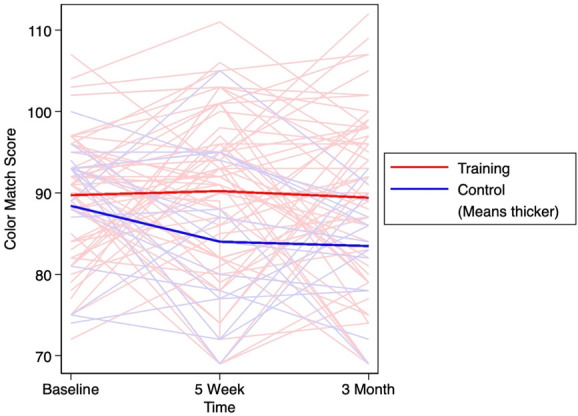
Color Match score over 3 months with thicker lines demonstrating the average score in the training and control groups.

Table 2 presents 5-week and 3-month changes in NCPT scores, including the average score for the battery of neurocognitive tests, average score for NCPT flexibility subscores, and the computerized Color Match (CM) score. In each group and both time points, the NCPT and flexibility NCPT scores increased, indicating improved neurocognitive performance. The effect size for the changes in NCPT score between the training and control group was small for both 5 weeks (*d* = 0.37) and for 3 months (*d* = 0.18). For the change in flexibility NCPT performance, the effect sizes were small for both 5 weeks (*d* = 0.30) and 3 months (*d* = 0.44). Higher scores on the computerized CM assessment also indicate a better performance. The computerized CM scores increased slightly (Δ = 0.6) among the training group at 5 weeks; however, it decreased slightly (Δ = −0.2) at 3 months in this group. In the control group, the computerized CM score decreased at both time points (5-week Δ = −4.4; 3-month Δ = −4.9). Small effect sizes were seen for 5-week (*d* = 0.44) and 3-month (*d* = 0.46) changes in computerized CM scores. The changes in NCPT score, NCPT flexibility subscore, and computerized CM are outlined in Figures 2–4 respectively. The 5-week and 3-month changes for each of the neurocognitive tests are provided in Appendix A, http://links.lww.com/PR9/A214.

**Table 2:** Change over 5 weeks and 3 months in cognitive training scores for training and control groups.

	5-wk change			3-mo change		
	Training	Control	Effect size	Training	Control	Effect size
Overall NCPT	**3.9 (6.0) [2.4, 5.4]**	**2.1 (3.3) [0.7, 3.5]**	0.37	**2.9 (4.8) [1.5, 4.4]**	2.1 (4.3) [−0.0, 4.2]	0.18
Flexibility NCPT	**3.7 (6.5) [2.1, 5.3]**	1.9 (5.3) [−0.4, 4.1]	0.30	**3.7 (6.4) [1.7, 5.6]**	0.9 (6.4) [−2.3, 4.1]	0.44
Computerized Color Match score	0.6 (11.6) [−2.5, 3.6]	−4.4 (11.1) [−10.6, 1.8]	0.44	−0.2 (11.4) [−3.2, 2.9]	−4.9 (9.2) [−10.0, 0.2]	0.46

Bold indicates *P* < 0.05.

NCPT, neurocognitive performance test.

**Figure 5.**
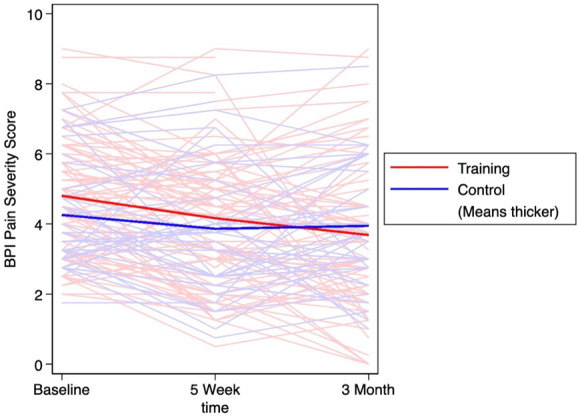
BPI pain severity score over 3 months with thicker lines demonstrating the average score in the training and control groups. BPI, brief pain inventory.

**Figure 6.**
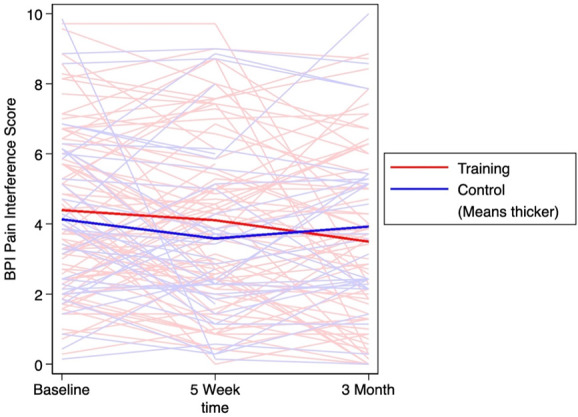
BPI interference score over 3 months with thicker lines demonstrating the average score in the training and control groups. BPI, brief pain inventory.

Table 3 presents the 5-week and 3-month change from baseline in BPI severity and interference scores by group. For both groups, the pain scores numerically decreased at follow-up. Effect size calculations between the control and training groups were *d* = 0.16 for the 5-week change in BPI severity and *d* = 0.39 effect for the 3-month change. The effect size for change in BPI interference between the groups was *d* = 0.09 for the 5-week and *d* = 0.20 for the 3-month change. The changes in BPI severity scores over 3 months are outlined in Figure 5, and the corresponding changes in BPI interference scores in Figure 6.

**Table 3:** Change over 5 weeks and 3 months in pain scores for training and control groups.

	5-wk change			3-mo change		
	Training	Control	Effect size	Training	Control	Effect size
BPI severity	**−0.6 (1.5) [−1.0, −0.3]**	−0.4 (1.5) [−0.9, 0.1]	0.16	**−1.0 (2.0) [−1.5, −0.5]**	−0.3 (1.6) [−0.8, 0.3]	0.39
BPI interference	−0.3 (1.9) [−0.7, 0.1]	−0.5 (2.4) [−1.4, 0.3]	0.09	**−0.7 (2.4) [−1.3, −0.2]**	−0.2 (2.5) [−1.1, 0.7]	0.20

Bold indicates *P* < 0.05.

BPI, brief pain inventory.

Table 4 presents the percentage of patients in each group who achieved a 30% and 50% reduction in BPI pain severity and pain interference scores from baseline to 3 months. The NNT for each group is provided. A greater proportion of patients in the training group reported a 30% or 50% reduction in pain severity and interference from baseline to 3 months. The difference was greatest for pain interference, with 43% of the training group reporting a 30% reduction at 3 months compared with 19% in the control group.

**Table 4:** Reduction in pain from baseline to 3 months.

	Training	Control	Number needed to treat
	Percentage	Percentage	
BPI pain severity score			
30% reduction	40.6	31.4	10.9
50% reduction	29.0	8.6	4.9
BPI pain interference score			
30% reduction	42.9	19.4	4.3
50% reduction	28.6	12.9	6.4

BPI, brief pain inventory.

We performed a subanalysis of primary and secondary outcome results stratified by adherence to the prescribed neurocognitive training intervention. Table 5 displays follow-up results for participants in the training group stratified by the level of adherence. Adherence level indicates whether patients participated in cognitive exercises for at least 980 minutes (≥80% of the expected 1,225 minutes in total) over the study period.

**Table 5:** Change over time by adherence (≥980 minutes).

	Change over time by adherence (≥980 minutes) (n = 10)			
	5-wk change	Paired *t*-test *P*	3-mo change	Paired *t*-test *P*
NCPT				
All	3.9	**<0.001**	2.9	**<0.001**
≥80% training adherence	7.3	**0.003**	3.7	0.127
<80% training adherence	3.3	**<0.001**	2.8	**<0.001**
Flexibility NCPT				
All	3.6	**<0.001**	3.7	**<0.001**
≥80% training adherence	4.5	0.054	2.6	0.511
<80% training adherence	3.5	**<0.001**	3.9	**<0.001**
Color Match				
All	0.9	0.574	0.0	0.991
≥80% training adherence	1.4	0.705	4.9	0.296
<80% training adherence	0.8	0.642	−0.7	0.677
BPI severity				
All	−0.7	**<0.001**	−1.0	**<0.001**
≥80% training adherence	−1.7	**0.004**	−1.6	**0.039**
<80% training adherence	−0.5	**0.012**	−1.0	**<0.001**
BPI interference				
All	−0.3	0.207	−0.7	**0.014**
≥80% training adherence	−1.9	**0.017**	−1.8	0.081
<80% training adherence	0.0	0.908	−0.6	0.070

Bold indicates *P* < 0.05.

BPI, brief pain inventory; NCPT, neurocognitive performance test.

The number of high-adherence participants was 10; this group attained better numerical results on 9 out of 10 measures across 5-week and 3-month follow-up (except NCPT flexibility subscore at 3 months).

## 4. Discussion

The aim of this study was to investigate the effects of a neurocognitive training program aimed at improving cognitive flexibility and self-reported pain parameters for patients with chronic hip, knee, and low back pain. We found evidence to support modest improvement in global NCPT and cognitive flexibility following a 5-week intervention. This study provided effect size estimates of the intervention on various neurocognitive performance measures. The effect sizes were small for total NCPT (5 weeks: *d* = 0.37; 3 months: *d* = 0.18) and for NCPT flexibility subscore (5 weeks: *d* = 0.30; 3 months: *d* = 0.44) but overall suggested that the 5-week intervention program can lead to improvement in neurocognitive performance that may last up to 3 months. In comparison with other studies measuring the impact of cognitive training interventions using the Lumosity platform, these effect sizes are similar to the change in cognition (*d* = 0.43) observed following 12 weeks of the four 30-minute Lumosity training sessions per week (vs crossword puzzles) in individuals with mild cognitive impairment.^31^ However, the effects sizes were smaller than those found for change in cognitive flexibility among 41 chemotherapy-treated breast cancer survivors who used the Lumos platform and a control group (*d* = 0.58).^43^ In this study, the participants engaged in four 20- to 30-minute sessions weekly over 12 weeks compared with this study with daily 35-minute cognitive training for 5 weeks. Our smaller effect sizes could be attributable to shorter training period but could also be related to population differences because their study included only women survivors of breast cancer with a minimum age of 40 years. In addition, participants in that study demonstrated higher levels of adherence, which was supported by research staff contacting participants once per week with a reminder to complete the exercises. The average change in NCPT scores observed among training group participants in this study (+3.8 points at 5 weeks and +2.9 points at 3 months) were smaller than those observed in a randomized controlled trial (RCT) with 4725 participants using the same training program over 10 weeks (mean change = 5.2).^38^ The change in the present training group, however, was larger than that observed in the control group engaging in crossword puzzles in the previous RCT (mean change = 2.1). In addition to the large difference in sample size between this and our study, the higher change observed in NCPT scores after the intervention is likely a result of the longer intervention period (10 weeks vs 5 weeks in this study), which should be taken into consideration for designing future studies.

The study's second objective was to examine whether participation in the training program was associated with pain severity and pain interference in patients with chronic pain. At both time points, the training group reported a slightly greater decrease in pain severity than the control group, however, the effect size estimates were small for pain severity (Cohen *d* = 0.16–0.39) and very small for pain interference (*d* = 0.09–0.20). These effect sizes are similar to those observed in a feasibility study exploring the impact of cognitive therapy on pain severity (*d* = −0.13) and pain interference (*d* = −0.10) among individuals with spinal cord injury–related pain^66^ and slightly smaller than the effect size for pain interference (*d* = −0.3) in a pilot study of Internet-based pain coping skills training program for individuals with systemic lupus erythematosus.^2^ However, our effect size findings for pain severity and pain interference were much smaller, compared with those observed in a randomized, controlled, pilot trial comparing a meaning-centered pain coping skills training vs usual care for patients with metastatic cancer (pain severity *d* = −0.75; pain interference *d* = −0.82)^78^ and the effect sizes for pain intensity (*d* = 0.65) and pain interference (*d* = 0.70) for cognitive behavioral therapy for veterans with chronic pain.^51^

The minimally clinically important difference (MCID) for BPI subscales is not well characterized; however, a study seeking to estimate the MCID for the worst pain item on the BPI revealed that a one-category decrease is associated with an absolute value of change ranging from 0.56 to 3.16.^47^ This finding suggests that the changes we observed in the training group (−0.3 to −1.0) may fall within the clinically meaningful range. Because the minimum clinically meaningful magnitude of change on the BPI scale may be controversial, we calculated the proportion of patients who achieved acceptable metrics of meaningful pain relief, that is, 30% or more and 50% or more. From baseline to 3 months, a greater proportion of individuals in the training group experienced 30% to 50% reductions in pain severity and pain interference compared with the control group. This information was used to calculate the NNT, which ranged from 4.3 for a 30% reduction in pain interference to 10.9 for a 30% reduction in pain severity. These are similar to NNT results reported in both pharmacological^64^ and nonpharmacologic studies^45,50,64,72^ on pain, although slightly higher than the NNT found in a study on CBT (2.3–5.2) for chronic pain.^30,72^ Although our study was underpowered to analyze pain outcomes data, the low-risk nature of the intervention warrants further evaluation and larger clinical trials assessing efficacy as a pain management strategy. As the spaghetti plots demonstrate, despite the overall minor decrease in BPI severity and interference scores, there is marked variability among participants, including directional changes in the slopes of the scores from baseline to 5 weeks vs 5 weeks to 3 months of follow-up. It is likely that some of the interaction can be attributed to regression to the mean, considering the variability in pain scores over time in patients with low back or joint pain.^9,54,56,60,61,67,69^

We observed trends toward improvement in cognitive scores, including flexibility scores, and improvement in pain severity and interference. However, we cannot conclude whether the change in pain scores was driven by changes in cognitive function in general or cognitive flexibility. The ad hoc analyses we performed based on adherence rates may help clarify this point. The examination of the difference between individuals in the intervention group indicated that those reaching ≥80% of the training goal (in overall minutes trained over 5 weeks) achieved a modestly higher magnitude of changes in cognition and pain parameters, compared with those with low adherence; however, these effects were small and not consistently seen across groups.

There was a low percentage of participants who withdrew from the study or did not complete follow-up (13%). On average, participants in the intervention group completed 55% of the prescribed intervention regimen (679 ± 297 minutes). Nearly 74% of participants did participate in 80% of training days, but only 13% of patients achieved high adherence in terms of overall minutes trained, which limits the reliability of the results and necessitates an improvement in the feasibility and implementation of the intervention. Although it is feasible to deliver neurocognitive training interventions in this setting, the training program needs to be adapted to improve adherence. Adaptations should account for the unique considerations of populations experiencing chronic pain, where it is also important to consider minimizing factors, such as fatigue or pain, that may be exacerbated during long training sessions themselves.^13^ Furthermore, our study employed a computer-based intervention. Previous literature suggests that participants prefer mobile devices over computers given the ease with which they can be carried even in populations where mobile usage is low.^1^ It is possible that individuals with chronic pain may prefer the convenience and physical comfort of a mobile phone that they can use in different positions.

Our findings should be interpreted in light of several study limitations. First, the data for this study were collected from a single center, limiting the generalizability of the results. We employed a passive control group, which may limit the internal validity of the results.^12,52^ Because the study was not blinded and did not have an active control group, it is possible that the interaction/study participation alone may be responsible for a change in pain scores and that another computer training activity (eg, playing a computer game) may offer the same result. Our objective was to conduct a proof-of-concept trial to assess preliminary signal of treatment efficacy of the neurocognitive training for chronic pain. Future research should consider a comparative effectiveness design evaluating neurocognitive performance and pain scores in groups participating in a cognitive training program with participation in a computer game that was not designed to improve cognitive flexibility.

The majority of the modules included in the training are described by the Lumosity platform as task-switching exercises. The current article sought to expand on a previous study^5^ that measured cognitive flexibility using task-switching exercises (TMT A and TMT B) in surgery patients to predict chronic pain and similarly used these exercises in addition to the other training modules provided by the platform. However, this narrow focus does not account for all the various cognitive mechanisms involved in cognitive flexibility, and therefore, the interpretation of results should be limited to this conceptualization of cognitive flexibility.

## 5. Future directions

Neurocognitive training interventions have been tested in various settings, including with patients with posttraumatic stress disorder,^11,15,42,46,57,58,62^ delirium,^53^ and traumatic brain injury.^18,24,42,58^ These and the present study suggest that an important next step for researchers is to determine which specific type of cognitive training programs are effective for which populations and the ideal timing, mode of delivery, and dosage/intensity.

Given that impairment in cognitive flexibility is associated with persistent pain after surgery, it may be reasonable to implement similar interventions in the perioperative setting. Changing the setting of the intervention will require adaptations to meet patient needs.^40^ Notably, results of a qualitative study suggest that surgery patients are willing to engage in such an intervention after surgery; however, they reported several potential barriers, including fatigue, cognitive overload, and lack of familiarity with technology.^40^ These findings combined with those from this study should be considered when adapting the intervention for those with chronic pain. Furthermore, although a larger trial is necessary to provide reliable evidence on the use of a neurocognitive training program to mitigate chronic pain, given the understanding of the effect sizes of this intervention (both on cognition and pain) as a result of this study, it would be now possible to plan the next properly powered clinical trial to test both the effect of the intervention (ensuring appropriate adherence) and formal mediation analysis to determine whether the improvement in neurocognitive measures is independently associated with improvement in pain.

## 6. Conclusion

Our findings suggest that participating in a neurocognitive training program may improve overall cognitive performance and cognitive flexibility over 3 months in individuals with chronic pain. In addition, engaging in such a training program may reduce pain severity. If our findings are subsequently validated in a larger, prospective, perioperative study, this research might result in the development of an implementable strategy to treat chronic musculoskeletal pain. In addition, because impairment in cognitive flexibility is associated with higher risk of chronic pain after surgery, there is a potential to implement this approach perioperatively to prevent and mitigate chronic postsurgical pain and improve postoperative functioning and quality of life in adults undergoing surgical procedures. It is critical that future research addresses barriers and facilitators to the feasibility of implementing such interventions and determines the optimal delivery method, intensity, and duration of neurocognitive interventions for the treatment and the prevention of chronic pain.

## Disclosures

Lumos Labs kindly provided the neurocognitive training modules and training accounts for participants. No other funding sources were provided. The authors declare no conflicts of interest.

## Appendix A. Supplemental digital content

Supplemental digital content associated with this article can be found online at http://links.lww.com/PR9/A214.
